# Supplementary material for: Cow and Human Milk-Derived Exosomes Ameliorate Colitis in DSS Murine Model
Source: Nutrients. 2020 Aug 26;12(9):2589. doi: 10.3390/nu12092589 (PMC7551078; doi:10.3390/nu12092589)
Supplement: Supplementary file 1 [file nutrients-12-02589-s001.zip › Suplementary data.docx]

**Figure S1 Western blot**

**S1A**: Protein expression of CD81 (exosome-related protein) and HSP70 (non exosomal protein). CD81 is expressed in MDE and breast cells, HSP70 is only expressed in breast cells and not detected in MDE.

**S1B**: TGF-β1 expression in MDEs (pellet 135K) compared with the pellet following ultracentrifugation at 40 K

**Table S1-Primers mRNA**

| *DNMT1* (mouse) | For 5’-CCTAGTTCCGTGGCTACGAGGAGAA |
| --- | --- |
|  | Rev 5’-TCTCTCTCCTCTGCAGCCGACTCA |
| *DNMT3a* (mouse) | For 5’-GCCGAATTGTGTCTTGGTGGATGACA |
|  | Rev 5’-CCTGGTGGAATGCACTGC AGAAGGA |
| *TNF*-*α* (mouse) | For 5’-GTTCTGTCCCTTTCACTCAC |
|  | Rev 5’-TGCCTCTTCTGCCAGTTC |
| *IL-6* (mouse) | For 5’- GAGTCACAGAAGGAGTGGCTAAGGA |
|  | Rev 5’-CGCACTAGGTTTGCCGAGTAGATCT |
| *GAPDH* (mouse) | For 5’-GCCTTCCGTGTTCCTACC |
|  | Rev 5’-CTTCACCACCTTCTTGATGTC |
| *TBP* (mouse) | For 5’-ACCGTGAATCTTGGCTGTAAAC |
|  | For 5’-GCAGCAAATCGCTTGGGATTA |

﻿
